# Supplementary material for: Nanoenzyme Reactor-Based Oxidation-Induced Reaction for Quantitative SERS Analysis of Food Antiseptics
Source: Biosensors (Basel). 2022 Nov 8;12(11):988. doi: 10.3390/bios12110988 (PMC9688296; doi:10.3390/bios12110988)
Supplement: Supplementary file 1 [file biosensors-12-00988-s001.zip › biosensors-1964131-supplementary.pdf]

Nanoenzyme Reactor-Based Oxidation-Induced Reaction for Quantitative SERS Analysis of Food Antiseptics

Linmin Chen [1], Meihuang Zeng [1], Jingwen Jin [2], Qiuhong Yao [2], Tingxiu Ye [3], Longjie You [4], Xi Chen [5,*], Xiaomei Chen [1,*] and Zhiyong Guo [2,6,*]

[1] College of Ocean Food and Biological Engineering, Jimei University, Xiamen 361021, China
[2] Institute of Analytical Technology and Smart Instruments and College of Environment and Public Healthy, Xiamen Huaxia University, Xiamen 361024, China
[3] College of Pharmacy, Xiamen Medicine College, Xiamen 361005, China
[4] National Quality Supervision and Inspection Center for Incense Products (Fujian), Quanzhou 362600, China
[5] State Key Laboratory of Marine Environmental Science, Xiamen University, Xiamen 361005, China
[6] Xiamen Environmental Monitoring Engineering Technology Research Center, Xiamen 361024, China
* Correspondence: xichen@xmu.edu.cn (X.C.); xmchen@jmu.edu.cn (X.C.); guozy@hxxy.edu.cn (Z.G.)

Keywords: nanoenzyme reactor; oxidation-Induced Reaction; SERS; food antiseptics

Experimental Section

Pre-treatment of actual samples. The practical application of the Au@SNF NPs based nanoenzyme reactor was used to determine sorbic acid in various biscuit samples. The food samples of solid biscuits were tested to verify the practical application of the method. The food samples were prepared according to the Chinese national standard method (GB 5009.28-2016, Determination of benzoic acid, sorbic acid and sodium saccharin in food). Firstly, the solid sample was crushed until homogeneous. Secondly, 2 g of sample (accurate to 0.001 g) was taken and sonicated in 25 mL of deionised water at 50°C for 20 minutes, then cooled to room temperature. Then, 2 mL of zinc acetate solution (183 g/L) and 2 mL of potassium ferricyanide solution (92 g/L) were added and mixed, followed by centrifugation at 8000 r/min for 5 min. After that, 20 mL of water was added to the residue, mixed by vortexing, and sonicated for 5 min, after which the residue was centrifuged at 8000 r/min for 5 min. Finally, all supernatants were transferred to the same 50 mL volumetric flask and fixed to the scale with water, mixed and filtered through a 0.22 ◎m membrane and stored in the refrigerator until measured.

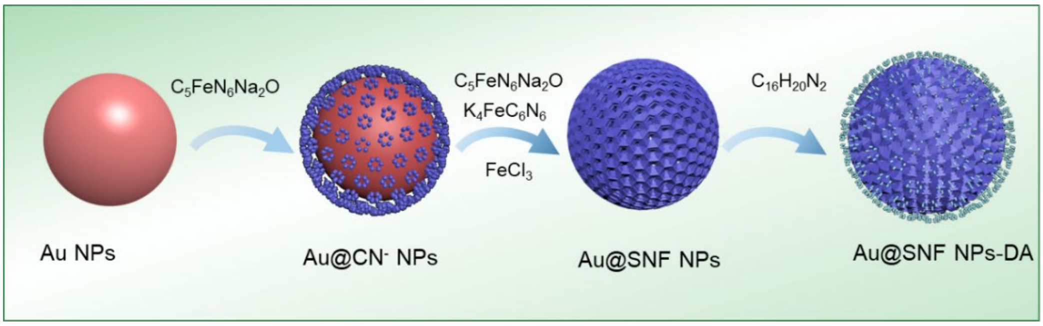

Figure S1. Schematic diagram of the nanoenzyme preparation process

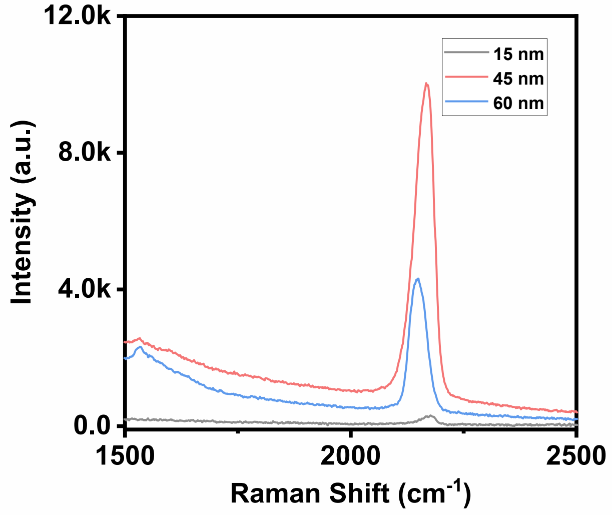

Figure S2. Optimization of the particle size of gold nanoparticles

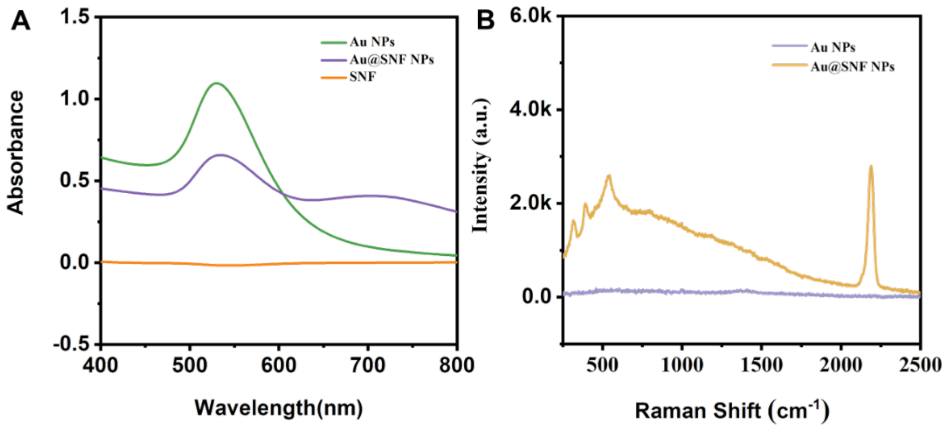

Figure S3. (A) UV-Vis spectral behaviors of Au NPs, Au@SNF NPs, SNF; (B) Raman spectra of Au NPs and Au@SNF NPs

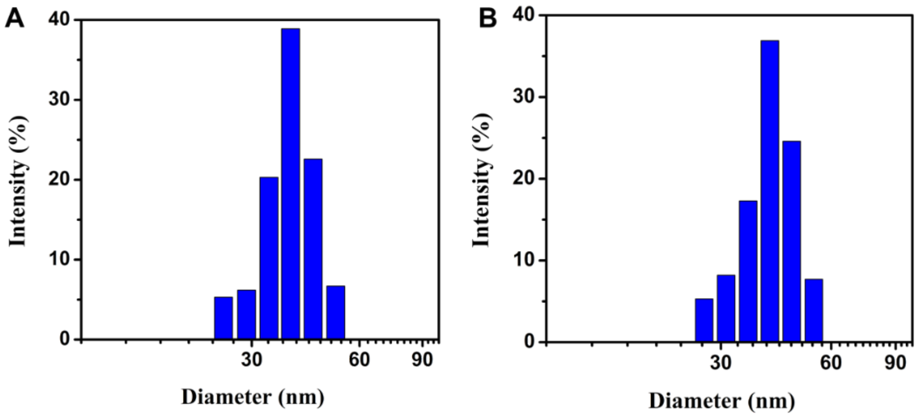

Figure S4. The size of (A) Au NPs, (B) Au@SNF NPs by the DLS studies

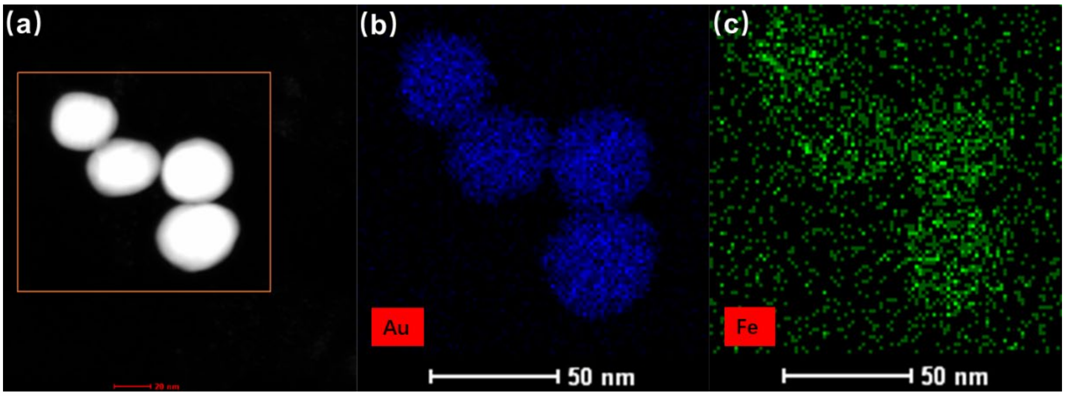

Figure S5. The HRTEM images and STEM-EDS elemental analysis of Au@SNF NPs

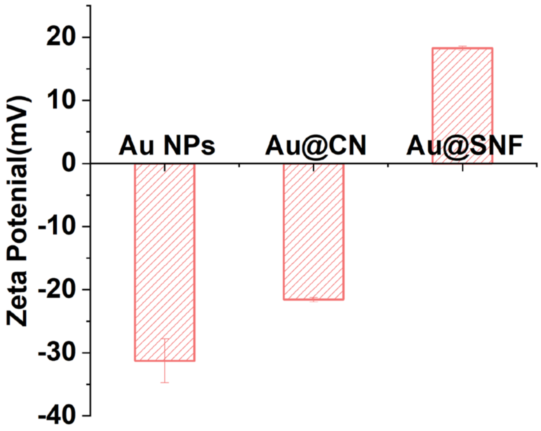

Figure S6. Zeta potentials of Au NPs, Au@SNF NPs, Au@CNF NPs

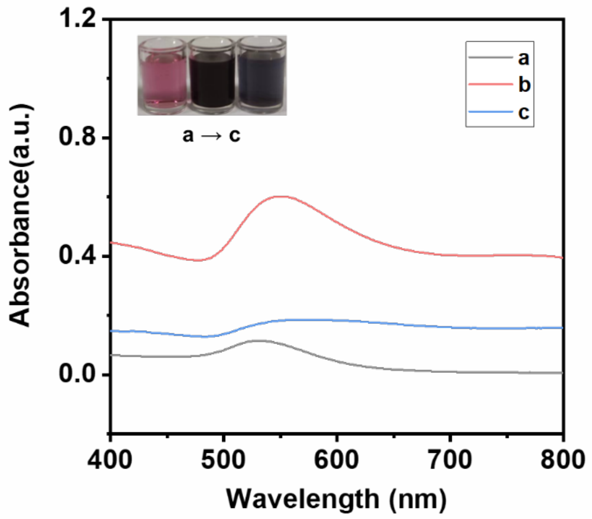

Figure S7. The UV-vis absorbance spectrum and colour of different nanomaterials: (a) Au NPs, (b) Au@SNF NPs, (c) Au@SNF NPs-DA

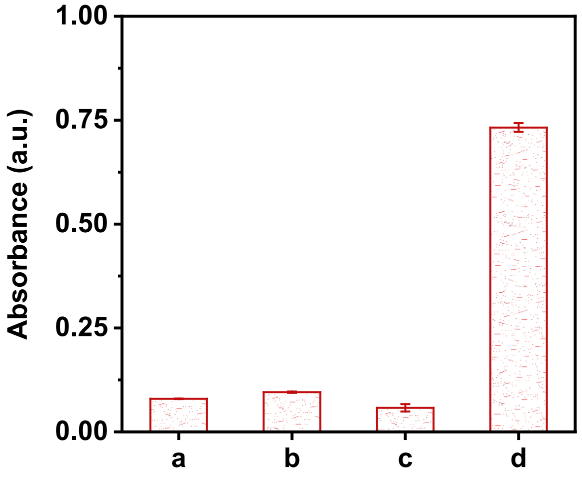

Figure S8. The UV-Vis absorbance at 652 nm of the reaction systems with different components.

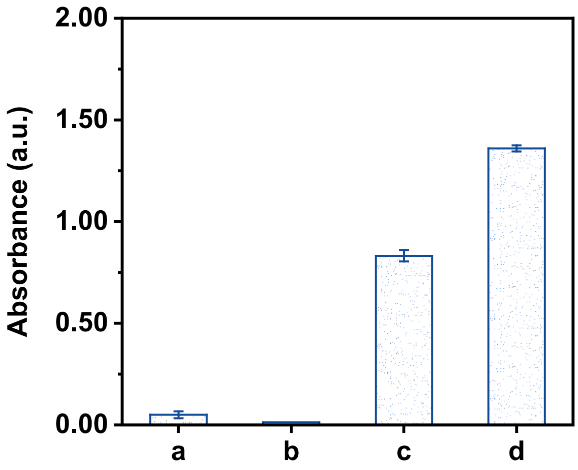

Figure S9. The UV-Vis absorbance at 652 nm of the Fe^{3+}-H_2O_2 -Fenton-like reaction with different components.

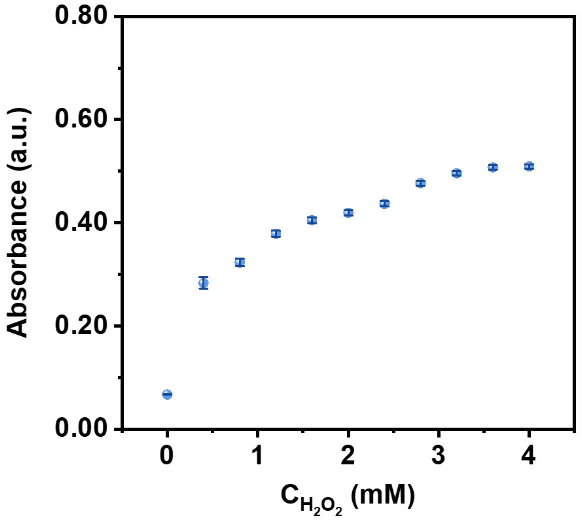

Figure S10. The steady-state kinetic assay of Au@SNF NPs-catalyzed decomposition of H_2O_2.

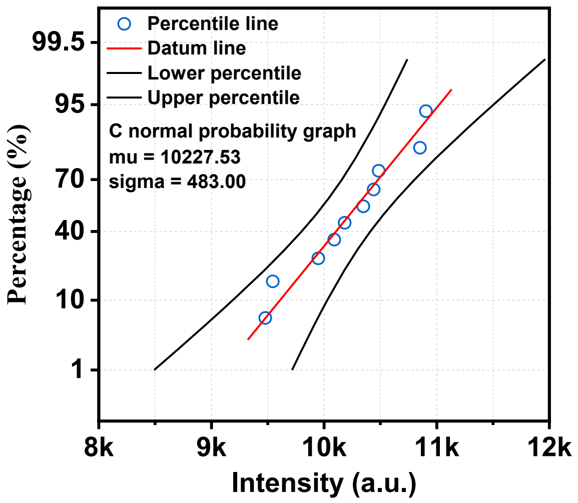

Figure S11. The SERS response of 2185 cm^{-1} to Au@SNF NP with ten times.

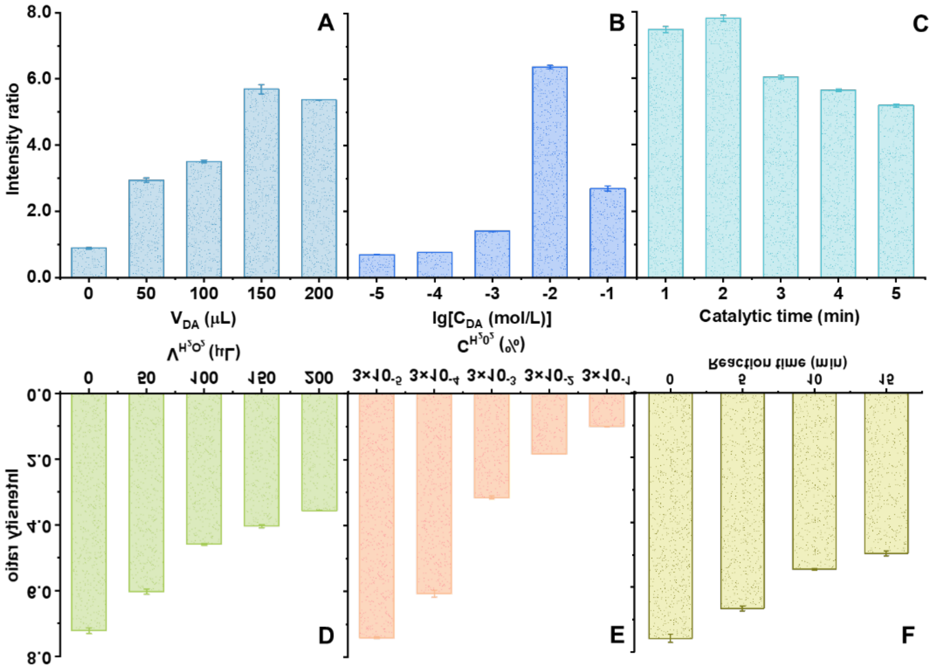

Figure S12. (A) Dopamine volume, (B) dopamine concentration, (C) catalytic time (D) H_2O_2 volume (E) H_2O_2 concentration (F) reaction time, effects on the Raman spectral signal of the nanoenzyme reactor.

Table S1. Comparison of the analytical performance of various determination methods for sorbic acid

Methods	Linearity range （mg L^{-1}）	Limit of detection （mg L^{-1}）	recovery percentages	Reference
HS-SPME	5-150	1.1-1.7	92-106%	[26]
SERS	0.0066-1.59	0.0032	95.8-104%	[31]
AA-DLLME-OPS	0.05-100	0.02	96-101%	[32]
SPE	1-45	0.502	81-96%	[33]
LVSS-PS-CDEKC	0.5-0.01	0.001	97-102%	[34]
SERS	0.000112-1.12	1.344×10^{-5}	90.24-109.40%	This work

[26] Tungkijanansin N, Alahmad W, Nhujak T, et al. Simultaneous determination of benzoic acid, sorbic acid, and propionic acid in fermented food by headspace solid-phase microextraction followed by GC-FID. Food Chemistry. 2020, 329: 127161. 10.1016/j.foodchem.2020.127161.

[31] Luo Y, Jing Q, Li C, et al. Simple and sensitive SERS quantitative analysis of sorbic acid in highly active gold nanosol substrate. Sensors and Actuators: B. Chemical. 2018, 255: 3187-3193. 10.1016/j.snb.2017.09.144.

[32] Timofeeva I, Kanashina D, Stepanova K, et al. A simple and highly-available microextraction of benzoic and sorbic acids in beverages and soy sauce samples for high performance liquid chromatography with ultraviolet detection. Journal of Chromatography A, 2019, 1588: 1-7. 10.1016/j.chroma.2018.12.030.

[33] Kefi B B, Baccouri S, Torkhani R, et al. Application of Response Surface Methodology to Optimize Solid-Phase Extraction of Benzoic Acid and Sorbic Acid from Food Drinks. Foods, 2022, 11(9): 1257. 10.3390/foods11091257.

[34] Pieckowski M, Kowalski P, Bączek T. Combination of large volume sample stacking with polarity switching and cyclodextrin electrokinetic chromatography (LVSS-PS-CDEKC) for the determination of selected preservatives in pharmaceuticals. Talanta, 2020, 211: 120673.

10.1016/j.talanta.2019.120673.
